# *In silico* targeting of colony-stimulating factor-1 receptor: delineating immunotherapy in cancer

**DOI:** 10.37349/etat.2023.00164

**Published:** 2023-08-31

**Authors:** Zahra Azhar, Richard P. Grose, Afsheen Raza, Zohaib Raza

**Affiliations:** University of Southampton, UK; ^1^Centre of Tumour Biology, Barts Cancer Institute, Queen Mary University of London, EC1M 6BQ London, UK; ^2^Department of Biomedical Sciences, College of Health Sciences, Abu Dhabi University, Abu Dhabi 59911, United Arab Emirates; ^3^Department of Chemistry, The University of Adelaide, 5005 Adelaide, South Australia, Australia

**Keywords:** Cancer drug development, tyrosine kinase inhibitors, drug resistance, computational modelling, molecular docking, target identification, lead optimization, targeted cancer therapy

## Abstract

**Aim:**

Delineate structure-based inhibition of colony-stimulating factor-1 receptor (CSF1R) by small molecule CSF1R inhibitors in clinical development for target identification and potential lead optimization in cancer therapeutics since CSF1R is a novel predictive biomarker for immunotherapy in cancer.

**Methods:**

Compounds were *in silico* modelled by induced fit docking protocol in a molecular operating environment (MOE, MOE.v.2015). The 3-dimensional (3D) X-ray crystallized structure of CSF1R kinase (Protein Databank, ID 4R7H) was obtained from Research Collaboratory for Structural Bioinformatics (RSCB) Protein Databank. The 3D conformers of edicotinib, DCC-3014, ARRY-382, BLZ-945, chiauranib, dovitinib, and sorafenib were obtained from PubChem Database. These structures were modelled in Amber10:EHT molecular force field, and quick prep application was used to correct and optimize the structures for missing residues, H-counts, termini capping, and alternates. The binding site was defined within the vicinity of the co-crystallized ligand of CSF1R kinase. The compounds were docked by the triangular matcher placement method and ranked by the London dG scoring function. The docked poses were further refined by the induced fit method. The pose with the lowest binding score (ΔG) was used to model the ligand interaction profile in Discovery Studio Visualizer v17.2. The co-crystallized ligand was docked in its apo conformation, and root-mean-square deviation was computed to validate the docking protocol.

**Results:**

All 7 CSF1R inhibitors interact with residue Met637 exhibiting selectivity except for edicotinib. The inhibitors maintain CSF1R in an auto-inhibitory conformation by interacting with Asp797 of the Asp-Phe-Gly (DFG) motif and/or hindering the conserved salt bridge formed between Glu633 and Lys616 thus stabilizing the activation loop, or interacting with tryptophan residue (Trp550) in the juxtamembrane domain. DCC-3014, ARRY-382, BLZ-945, and sorafenib bind with the lowest binding energy with CSF1R kinase.

**Conclusions:**

Pyrimidines are potent inhibitors that interact with CSF1R residues. DCC-3014 and ARRY-382 exhibit exceptional pharmaceutical potential exhibiting great structural stability and affinity.

## Introduction

Identification of genetic mutations underlying various subtypes of cancer has been an epochal turning point for cancer therapy [1]. The evolution of molecular taxonomy, based on genome-driven subtyping, or stratifying cancer according to molecular mapping has allowed rational drug design [2]. The development of targeted lead compounds has been optimized by the growth in big-data predictions as well as modelling and artificial intelligence (AI)-inspired drug discovery [2]. Molecular docking simulation allows the prediction of interactions of lead compounds with 3-dimensional (3D) conformations of target receptors [3]. This in turn aids in deciphering target specificity, binding energies, protein interactions, and drug cellular potency as well as predicting drug resistance mechanisms [2].

**Figure 1:**
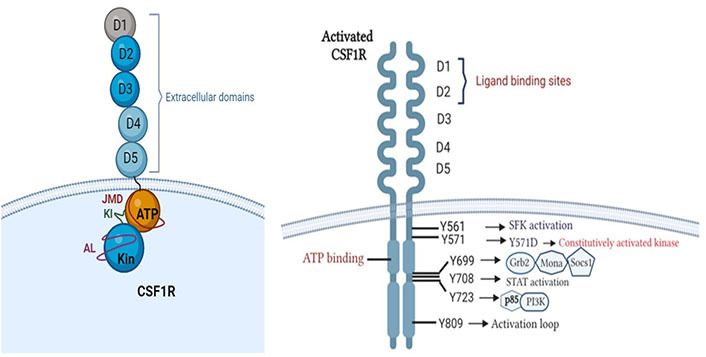
Structure of CSF1R. Phosphotyrosine residues of activated CSF1R initiating downstream signalling cascades (created with BioRender.com). Grb2: growth factor receptor-bound protein 2; Socs1: suppressor of cytokine signalling family protein 1; SFK: SRC-family protein tyrosine kinase; PI3K: phosphatidylinositol 3-kinase

The colony-stimulating factor-1 receptor (CSF1R/M-CSFR) is a tyrosine kinase receptor (RTKIII) located on the surface of macrophages, which is implicated with aberrant cell signalling and immunomodulation in cancer [4]. Canonically, CSF1R consists of an extracellular ligand binding domain having five immunoglobulin domains (D1–D5); a hydrophobic membrane-spanning region that connects with a split intracellular kinase domain consisting of a juxtamembrane domain (JMD); ATP binding site; tyrosine kinase domain with a kinase insert in between. The activation loop surrounds the kinase domain and plays a regulatory role in activation (Figure 1) [4–8].

Oncogenic variants arising due to translocations or oncogenic viral insertions result in conformational changes in CSF1R, increasing its binding affinity with ligand [colony stimulating factor-1 (CSF1)] or constitutively activating the receptor [8]. The activation is initiated by receptor dimerization and trans-autophosphorylation which generates phosphotyrosine residues [4, 9]. Each phosphotyrosine residue stimulates one of the downstream signalling cascades such as PI3K-Akt, mitogen-activated protein kinases (MAPK), SFK and signal transducer and activator of transcription (STAT) pathways [4, 8, 10, 11] as explained in Table 1. This results in abnormal and uncontrolled cellular proliferation, inhibition of apoptosis, cellular invasion, and metastasis, resulting in poor patient prognosis and outcomes [8, 12].

**Table 1:** Phosphotyrosine residues of CSF1R and their role in oncogenesis

**Phospho-tyrosine residue (CSF1R)**	**Mechanism of action**	**Cancer**	**Manifestation**	**References**
Y561	SFK activation	Lung, breast	Disruption of cell-cell adhesion via loss of E-cadherin resulting in anchorage-independent growth, motility and survival. DNA synthesis and cytoskeletal reorganization.	[13–20]
Y571	Kinase activation	HM (AML, aMPN)	Phosphorylation causes kinase activation. Mutated CSF1R-Y571D results in constitutively activated receptor.	[21, 22]
Y699	MAPK pathway	PTCL	Triggers the association of adapter proteins such as Grb2, Mona and Socs1 initiates monocyte differentiation. Cell proliferation.	[23]
Y708	STAT activation	FDC-P1 cell line	Mediates responses to interferons (IFNs), and activates macrophage cell proliferation.	[24, 25]
Y723	PI3K activation	Carcinoma	Mediates p85 subunit of PI3K association with CSF1R. Regulates adhesion, actin polymerization resulting in macrophage motility and invasion.	[9]
Y809	Tyrosine kinase activation	Fibroblasts	CSF1-induced autophosphorylation of CSF1R. Serves as a binding site for STAT proteins.	[10]

HM: hematological malignancies; AML: acute myeloid leukemia; aMPN: atypical myeloproliferative neoplasms; PTCL: peripheral T cell lymphoma; FDC-P1: factor dependent continuous-paterson 1

**Figure 2:**
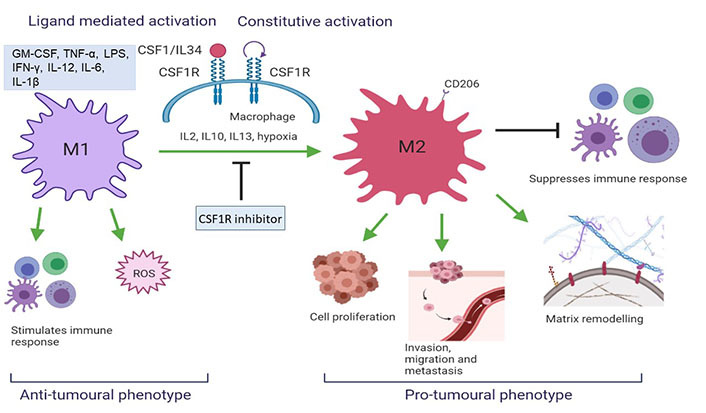
CSF1R activation results in a change in TAM phenotype. M2 macrophages initiate a pro-tumoral response which aggravates tumorigenesis whereas M1 initiates an anti-tumoral cascade (created with BioRender.com). ROS: reactive oxygen species; IL12: interleukin 12

CSF1R targeting via CSF1R inhibitors is widely studied in clinical trials [26]. Preclinical results show excellent *in vitro* and *in vivo* activity based on tumor-associated macrophages (TAMs) depletion, TAM reprogramming as well as inhibition of autocrine and paracrine signalling of supportive stromal cells within the tumour microenvironment (Figure 2) [8]. Early phase clinical trials show promising results of CSF1R inhibitors in tenosynovial giant cell tumours (TGCT), advanced solid tumours, pancreatic cancer, and gastrointestinal stromal tumour [27–30].

This paper discusses the modelling of interacting residues known as hotspots of CSF1R kinase on binding with its inhibitors, to understand the consequences of receptor inactivation and their impact on immunomodulation within tumour microenvironment.

## Materials and methods

Compounds were modelled *in silico* by induced fit docking protocol in the molecular operating environment (MOE, 2015.10). The 3D X-ray crystallized structure of CSF1R kinase (Protein Databank, ID 4R7H) was obtained from Research Collaboratory for Structural Bioinformatics (RSCB) Protein Databank [31]. The 3D conformers of edicotinib (CID 25230468) [32], DCC-3014 (CID 86267612) [33], ARRY-382 (SID 355048354) [34], BLZ945 (CID 46184986) [35], chiauranib (CID 49779393) [36], dovitinib (CID 135398510) [37] and sorafenib (CID 216239) [38] were obtained from the National Center for Biotechnology Information (NCBI)-RSCB PubChem Database. These structures were modelled in Amber10:EHT molecular force field and quick prep application was used to correct and optimize the structures for missing residues, H-counts, termini capping, and alternates. Protonate 3D application was used to optimize the protonation state to withstand molecular refinement of a docked pose. The binding site was defined within the vicinity of the co-crystallized ligand of CSF1R/FMS kinase. The compounds were docked by the triangular matcher placement method and ranked by the London dG scoring function. The docked poses were further refined by the induced fit method and scored by GBVI/WSA ΔG scoring function. The pose with the lowest binding score (ΔG) was used to model the ligand interaction profile in Discovery Studio Visualizer v17.2. The co-crystallized ligand was docked in its apo conformation, and root-mean-square deviation was computed to validate the docking protocol.

## Results

CSF1R kinase is a key target of small molecule CSF1R and multikinase inhibitors currently in clinical trials [39]. Kinases are dynamic proteins having a wide range of conformations governed by their activation state [2, 3]. The kinase domain has a bi-lobal kinase fold archetypal of protein kinase [3]. The conserved Asp-Phe-Gly (DFG) motif plays a regulatory role in catalysis, ATP, and substrate binding. It exists in two conformations depending on the activation state of the protein [3]. Activated kinase has a DFG-in conformation in which the aspartic acid is towards the B-phosphate of bound ATP to facilitate the catalytically significant Mg^2+^ ion; whereas phenylalanine points away from ATP, safely beneath the alpha-helix C [3]. In the DFG-out conformation, aspartic acid and phenylalanine switch places, hindering aspartic acid from interacting with ATP, and rendering the kinase inactive [3]. Structurally, tryptophan residue (Trp550) interacts with the carbonyl group of Asp796 which stabilizes the DFG-out conformation of the activation loop, thus maintaining CSF1R in a catalytically inactive state [3]. Moreover, some drugs act by breaking conserved salt bridges (Glu633 and Lys613), which are critical for catalytic activity [3].

Most Food and Drug Administration (FDA)-approved ATP-competitive small molecule inhibitors either bind to the catalytic active site or maintain an inactive enzyme conformation [3]. Since the CSF1R kinase domain has been implicated in CSF1R inhibition via pexidartinib (Protein Databank, ID 4R7H) [40], novel small molecule CSF1R inhibitors were docked within the same vicinity of the receptor. Pexidartinib has received FDA approval for advanced TGCT [41]. It forms hydrogen bonds with tyrosine residue (Tyr546) and Trp550 in the JMD of the kinase, whereas the pyridine nitrogen atom along with a network of hydrogen bonds, maintains an inactive state with a potent inhibitory concentration [half maximal inhibitory concentration (IC_50_) = 3 nmol/L] [41].

**Figure 3:**
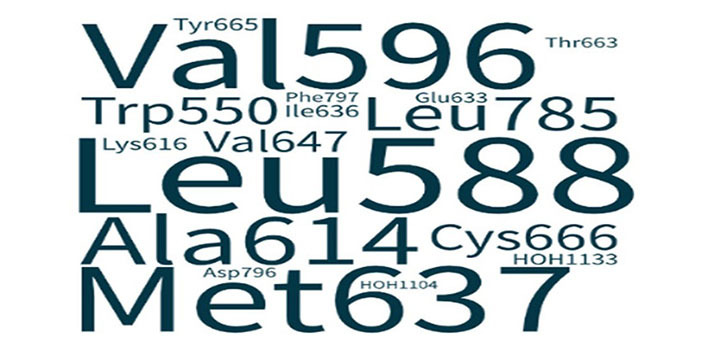
Word cloud illustrating the correlation of CSF1R amino acid residue and its interaction with CSF1R inhibitors. All 7 inhibitors interacted with Leu588 and subsequently with Met637 determining target specificity

This study identified mechanisms adopted for increased target specificity and cellular potency, which is highlighted in Table 2. The binding energies are mentioned in Table 3 which elucidates structural stability required in deriving pharmacokinetic parameters. Furthermore, the structural analysis of drugs interacting with CSF1R kinase delineated active amino acid binding sites of CSF1R as an approach towards relevant lead optimization, to reduce time wasted in identifying leads against intractable targets (Figure 3).

The word cloud illustrates the frequency of CSF1R residues targeted by drugs under study. Met637 and Leu588 are specificity markers of CSF1R and are shown as the largest within the word cloud ascertaining remarkable target specificity and precision therapy exhibited by the inhibitors under study. Subsequently, Val596, Ala614, Leu785, and Ile636 form a hydrophobic pocket which makes a favorable niche for DCC-3014 and dovitinib primarily, whereas, ARRY-382, sorafenib, and BLZ-945 interact with most of the residues of the hydrophobic pocket.

**Table 2:** Significant interacting residues of CSF1R and their functions

**Interacting binding sites**	**Functions**	**References**
Thr663	Gatekeeper residue	[26, 42]
Cys666	ATP binding site residue	[26]
Tyr546, Trp550	Regulates JMD to maintain an autoinhibitory state	[39]
Asp796	Form the DFG-motif, regulates the activation loop to maintain an autoinhibitory conformation	[8]
Met637, Leu588	Residues of CSF1R specificity	[26, 42]
Lys616, Glu633	Forms salt bridge regulating activation	[34]
Leu640, Ile646, Leu769, Leu785, Val596, Cys774, Ile794	Forms a hydrophobic pocket	[34]

**Table 3:** Binding energies (kcal/mol) of CSF1R inhibitors with CSF1R kinase

**CSF1R inhibitors**	**Binding energy ∆G (kcal/mol)**
DCC-3014	–10.64
ARRY-382	–10.26
Sorafenib	–10.24
BLZ-945	–9.58
Chiauranib	–8.49
Dovitinib	–8.38
Edicotinib	–7.35

## Discussion

DCC-3014, ARRY-382, BLZ-945, and sorafenib (multikinase inhibitor) bind CSF1R kinase with the lowest binding energies, exhibiting strong structural stability (Table 2) making them promising leads. Contrary to its fame in trials as a potent, oral brain penetrant CSF1R inhibitor, edicotinib exhibits weak binding at CSF1R kinase releasing a high binding energy. This warrants further investigation as it implicates an alternate mechanism of action. The CSF1R inhibitors with the lowest binding energies are the most stable [43] and thus, the most potent binders of the kinase domain of CSF1R.

**Figure 4:**
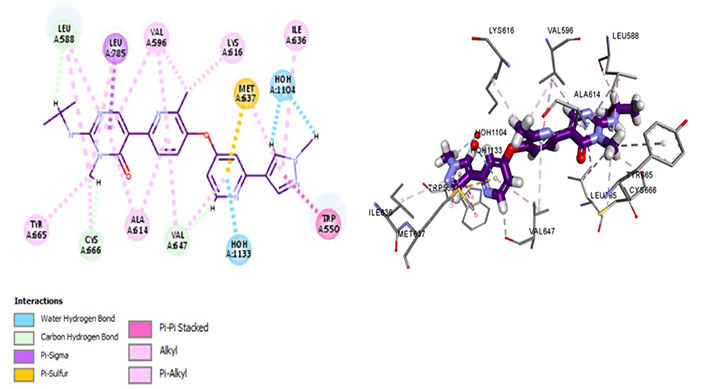
Docked structure of CSF1R kinase domain with small molecule multikinase inhibitor, DCC-3014 (left: 2D and right: 3D) in which interacting residues are depicted as balls colored according to the type of interaction. H: hydrogen

**Figure 5:**
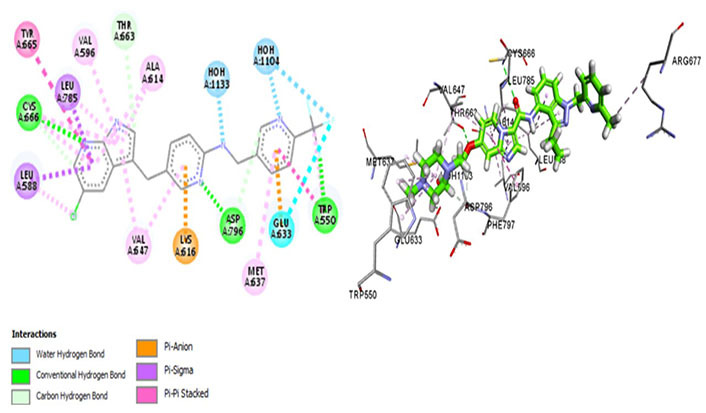
Docked structure of CSF1R kinase domain with small molecule multikinase inhibitor, ARRY-382 (left: 2D and right: 3D)

**Figure 6:**
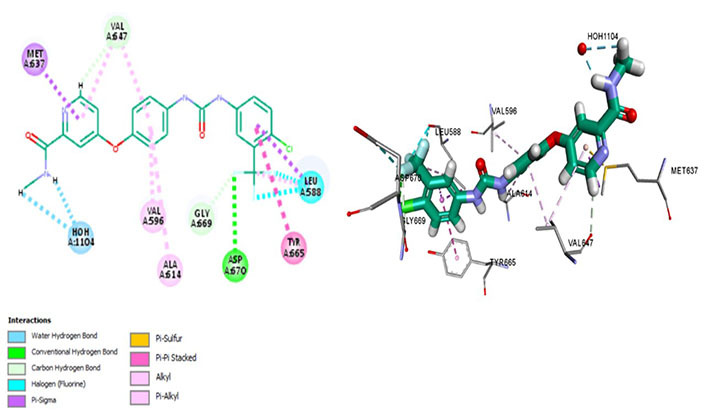
Docked structure of CSF1R kinase domain with small molecule multikinase inhibitor, sorafenib (left: 2D and right: 3D)

**Figure 7:**
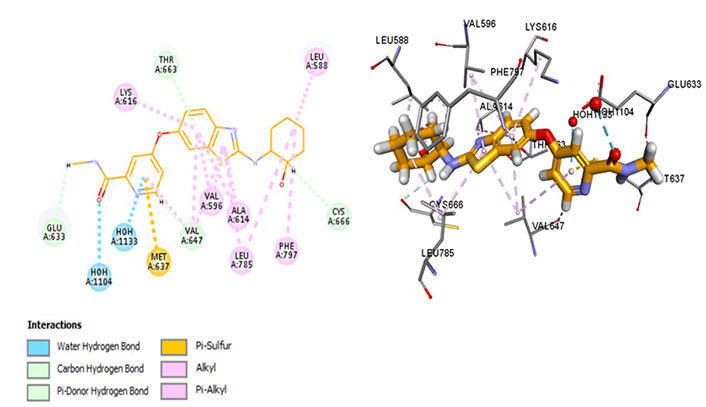
Docked structure of CSF1R kinase domain with small molecule multikinase inhibitor, BLZ-945 (left: 2D and right: 3D)

**Figure 8:**
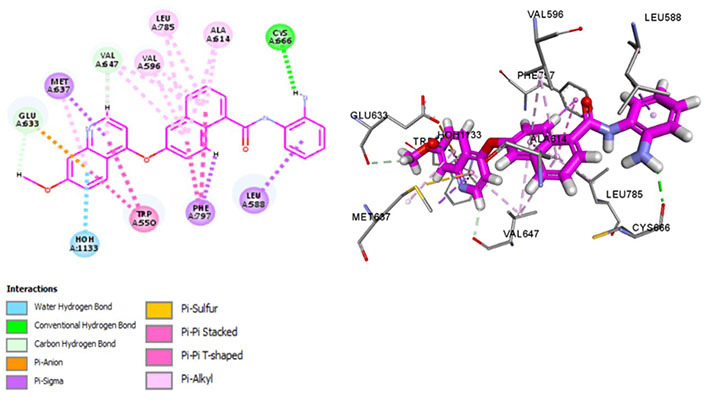
Docked structure of CSF1R kinase domain with small molecule multikinase inhibitor, chiauranib (left: 2D and right: 3D)

**Figure 9:**
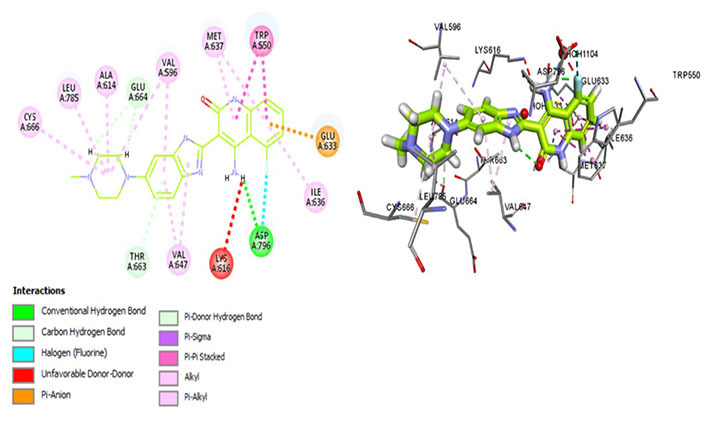
Docked structure of CSF1R kinase domain with small molecule multikinase inhibitor, dovitinib (left: 2D and right: 3D)

**Figure 10:**
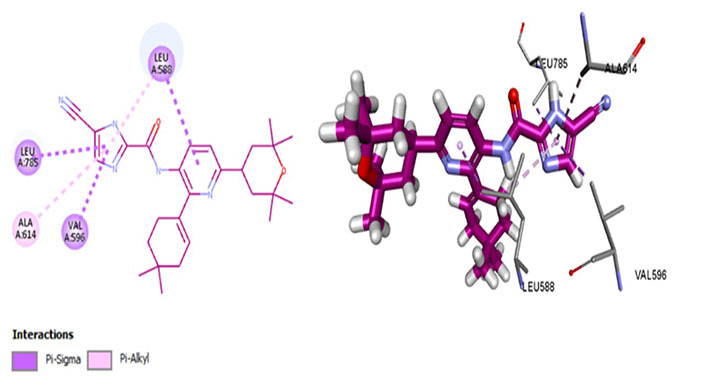
Docked structure of CSF1R kinase domain with small molecule multikinase inhibitor, edicotinib (left: 2D and right: 3D)

Evidence suggests quinolone derivatives, carboxamides, and pyrimidines are potent inhibitors [39] which are in concordance with the findings of the *in silico* study performed (Figures 4–10). The binding affinities varied and significant molecular interactions with essential residues have been highlighted as a strategic approach for targeted cancer drug development. Similarly, 7 CSF1R inhibitors were docked to analyze their molecular mechanism of action which has been described in detail below.

### DCC 3014: vimseltinib

TGCT is a morbid benign tumour that rarely metastasizes in lungs and lymph nodes resulting in death within a median of 21.5 months post-diagnosis of malignancy. Where pexidartinib has received FDA approval for TGCT, it is implicated with drug-limiting liver toxicity. This led to drug screening and the discovery of DCC-3014 which inhibits CSF1R with an IC_50_ of 3.7 nmol/L [44]. Human osteoclast precursor cells require CSF1 or receptor activator of nuclear factor kappa B ligand for differentiation [44]. In an *in vitro* assay, DCC-3014 blocked osteoclast differentiation and maturation with an IC_50_ of 9.3 nmol/L [44]. The drug exhibits a safe and efficacious pharmacokinetic profile. It is highly aqueous with minimal inhibition of drugs interacting with cytochrome P450 (CYP) enzymes or human Ether-à-go-go-Related Gene (hERG) channel, marking its stability in liver microsomes and low risk of QT interval (Q and T electrical rhythm of the heart) prolongation [44]. Proven pre-clinical efficacy has encouraged further investigation where it has shown a decline in the tumour burden of TGCT patients [45]. It disrupts autocrine and paracrine signalling between inflammatory and TGCT cells [45].

A dose escalation study for TGCT has shown 30 mg for 5 days as a loading dose with a repetition of 30 mg twice weekly in cycles of 28 days to decrease non-classical monocytes [45]. Symptomatic improvements were observed in terms of pain, swelling, and range of motion [45]. Vimseltinib is currently being evaluated in phase I/II as a single agent for TGCT and solid tumours and in combination with anti-programmed death ligand 1 (PD-L1) antibodies against advanced or metastatic sarcomas [45].

Amongst all 7 kinase inhibitors, DCC-3014 binds with the lowest binding energy, having the most stable conformation and proving to be the most promising lead compound in the venture of drug development (Figure 4). Named vimseltinib, findings of phase I clinical trials demonstrate it to be a well-tolerated oral drug in patients with advanced sarcoma and TGCT [44]. DCC-3014 interacts with Met637, exhibiting exceptional target specificity. The kinase selectivity pocket differs by a single residue at Met637 for CSF1R and Leu644 for tyrosine-protein kinase KIT (c-KIT) [14]. The drug targets the JMD and maintains the kinase in an autoinhibitory conformation by binding with Trp550 which stabilizes the activation loop in a DFG-out conformation [44]. Vimseltinib (DCC-3014) is thus termed a selective switch-control CSF1R inhibitor [44]. Moreover, it is not a substrate of the P-glycoprotein efflux pump as it strongly binds with Cys666 which is a residue of the ATP binding site, thus evading one of the most significant drug resistance mechanisms, making it a promising drug candidate against cancers overexpressing CSF1R.

### ARRY-382

ARRY-382 is being evaluated for ovarian cancer, triple-negative breast cancer, head and neck squamous cell cancer, bladder cancer, metastatic colorectal cancer, pancreatic ductal adenocarcinoma, gastric cancer, advanced unresectable melanoma, advanced PD-L1 positive non-small cell lung cancer (NSCLC). Having a maximum tolerated dose (MTD) of 300 mg in combination with pembrolizumab, the trial has reported limited clinical benefits in addition to patients experiencing dose-limiting toxicities exhibiting an increase in transaminases (10.5–83.3%) and increased creatine phosphokinase (18.2–50.0%) [46].

ARRY-382 exhibits promising pharmaceutical potential (IC_50_ = 9 nmol/L) [47] as it interacts with Cys666, Thr663, and Met637. Where Met637 interaction indicates high target specificity, strong interaction with Cys666 shows that it also evades the p-glycoprotein efflux pump. Thr663 is known to be a gatekeeper residue [26, 42]. The drug interacts with Trp550 as well as Tyr665, exhibiting similar efficacy as well as the mechanism of action of the prototypic CSF1R inhibitor—pexidartinib [39]. Additionally, it binds with Asp796 which stabilizes the DFG-out conformation of the activation loop, further stabilizing the autoinhibitory conformation of CSF1R kinase. The van der Waals forces are generated by the hydrophobic pocket formed by Val596, Ala614, Lys616, Val647, Thr663, and Leu785 [47]. The salt bridge is not conserved amongst Glu633 and Lys616, which causes CSF1R to be catalytically inactive (Figure 5).

### Sorafenib

Sorafenib interacts with Met637, exhibiting ligand-binding specificity. However, it maintains an autoinhibitory conformation by interacting with different residues forming a hydrophobic pocket of Val647, Val596, and Ala614. It forms a strong Pi-sigma bond with Leu588 and Met637 which are residues determining surface specificity. It regulates the activation loop to maintain CSF1R kinase in an autoinhibitory conformation by forming a conventional hydrogen bond with the Asp670. It evades the P-glycoprotein efflux pump, exhibiting great cellular potency, which is determined via its Pi-Pi interactions with Tyr665 (Figure 6).

Being a multikinase inhibitor, sorafenib inhibits cell proliferation as well as angiogenesis by inhibiting rapidly accelerated fibrosarcoma (RAF), vascular endothelial growth factor (VEGF), and platelet-derived growth factor (PDGF) pathways in addition to CSF1R. Associated with wide-ranging adverse effects, it showed 3 months longer median survival for hepatocellular carcinoma (HCC) than those given placebo [48], however, recent phase III trial has given camrelizumab (C) plus rivoceranib precedence over sorafenib for HCC based on significantly prolonged progression-free survival (PFS), overall survival (OS) and overall response rate (ORR) [49]. Nevertheless, it received FDA approval in 2006 for renal cell carcinoma after exhibiting a two-fold increase in PFS [50].

### BLZ-945

Sotuletinib (BLZ945) is a brain-penetrant, oral CSF1R inhibitor that has demonstrated a reduction in TAM recruitment, tumour growth, and resistance to programmed cell death 1 (PD-1) inhibitors in animal models of intracranial glioblastoma multiforme (GBM). Recommended phase II dose (RP2D) is 1,200 mg/day (4 days on/10 days off) for single-agent BLZ945. The MTD was 700 mg/day (4 days on/10 days off) for BLZ945 + spartalizumab (anti-PD-1 antibody) in patients having cancers with upregulated TAMs including GBM and pancreatic cancer [51]. Dose-limiting toxicities (DLTs) included elevated hepatic enzymes whereas grade 3 adverse events increased in the combination arm as opposed to monotherapy [51]. The drug causes TAM depletion concomitant with CD8^+^ infiltration which has been observed in breast cancer cell lines as well as cervical cancer cell lines [51].

The most potent CSF1R inhibitor BLZ-945 (IC_50_ = 1.2 nmol/L) exhibits carbon-hydrogen (C-H) interaction with gatekeeper residue Thr663 and ATP-binding site residue Cys666. An *in vivo* study demonstrated that CSF1R inhibitors show exceptional cellular potency [50% effective concentration (EC_50_) = 0.104–0.245 µmol/L] [52]. These drugs have a p-glycoprotein efflux ratio < 2, suggesting that these are not substrates of p-glycoprotein [multidrug resistance protein 1 (MDR-1)]. Therefore, the drug is being analyzed in a positron emission tomography (PET) imaging technique of CSF1R in the brain [52]. BLZ-945 forms a Pi-sulphur bond with Met637, highlighting its distinct selectivity and inhibitory potential. However, prolonged exposure has shown resistance that develops due to hyperactivation of PI3K which can be overcome using combination therapy [53]. Moreover, it interacts within the hydrophobic pocket formed by Val596, Ala614, Val647, Leu785, as well as Lys616. The Glu633 and Lys616 salt bridge is not conserved which maintains CSF1R in an inactive conformation. Moreover, it forms an alkyl bond with Phe797 of the DFG motif which stabilizes the activation loop in an inactive state (Figure 7).

### Chiauranib

Chiauranib inhibits tumor angiogenesis, tumor cell mitosis, and chronic inflammatory microenvironment by targeting angiogenesis-related kinases [VEGF receptor 2 (VEGFR2), VEGFR1, VEGFR3, PDGF receptor alpha (PDGFRα), and c-KIT], mitosis-related kinase Aurora B and chronic inflammation related kinase CSF1R [54]. Chiauranib binds with Met637 and Leu588, exhibiting specificity, and Cys666, depicting evasion of drug resistance via p-glycoprotein efflux pump. Exhibiting exceptional specificity, it has limited off-kinase effects demonstrating better clinical safety and efficacy against relapsed or refractory small cell lung cancer (SCLC). Currently, in the phase 3 trial for SCLC, the drug is being examined at an oral dose of 500 mg once daily, 21 days as a cycle until disease progression (NCT04830813). It interacts within the hydrophobic pocket formed by Val596, Phe797, Leu785, Glu633, and Val647 and maintains CSF1R kinase in a DFG-out conformation by binding with Phe797, stabilizing it in an autoinhibitory conformation. Pi-Pi interactions with Trp550 stabilize the activation loop in an inactive conformation, making it a potent inhibitor (Figure 8). Recent phase III trials suggest a single-digit nanomolar range of IC_50_ [55].

### Dovitinib

Dovitinib is a multi-kinase inhibitor that interacts with Met637, indicating its avidity for CSF1R. It forms a hydrogen bond with the activation loop residue Asp796, thus stabilizing the DFG-out conformation (Figure 9) which is essential for maintaining an autoinhibitory state of the protein. Moreover, interactions with Thr663 and Cys666 have been observed, indicative of its promising therapeutic potential in terms of exhibiting better cellular potency as well as evading resistance mechanisms. It exhibits similar interactions of the aforementioned kinase inhibitors forming a hydrophobic pocket by Leu785, Ala614, Val695, Val647, and Ile636. Moreover, the salt bridge interaction is hindered rendering the catalytic activity ineffective whereas the hydrophobic interaction aids prolonged drug-receptor binding.

Recent evidence shows dovitinib inhibits topoisomerase I and II by adjusting within the minor groove of the DNA and preventing decatenation elicited by topoisomerase [56]. The findings have been confirmed via molecular docking as well as DNA cleavage assays. Currently, in the phase III trial for renal cell carcinoma and phase II trial for breast cancer, dovitinib has shown promising potential for solid tumours with an IC_50_ of 36 nmol/L against CSF1R [57]. *In vitro* studies declared dovitinib as an intense anti-proliferative agent against glioma [57]. However, clinical trials have reported various adverse effects due to which the drug has not been FDA-approved yet.

### Edicotinib

Edicotinib (JNJ-40346527), chiauranib, and sorafenib are carboxamides that are in clinical trials for various cancers. Chiauranib and sorafenib are multikinase inhibitors whereas edicotinib is a selective CSF1R inhibitor. Surprisingly, edicotinib interacts via alkyl bonds (Figure 10) with no interaction with residues determining specificity (Met637) or therapeutic potential (Thr663, Cys666). Edicotinib has an IC_50_ of 3.2 nmol/L which is contrary to the results exhibited by molecular docking [58]. This suggests that the brain-penetrant CSF1R inhibitor edicotinib binds at a site other than the kinase domain of CSF1R warranting further investigation.

Targeted receptor inhibition and drug efficacy is determined via various pharmacokinetic factors including binding energy and IC_50_. Binding energy highlights drug-receptor binding, structural affinity, and/or lead-target conformational stability. It determines the strength of interaction between the lead molecule and target receptor, thereby playing an essential role in lead optimization and subsequently, drug development. It is calculated via various computational methods and requires apt simulations to derive accurate results.

IC_50_ is validated by *in vitro* methods using cell lines. Determining the correlation between *in silico* binding energies and IC_50_ values is governed by the accuracy of computational methods employed as well as cell line authenticity. Generally, a strong correlation between IC_50_ and binding energies is suggestive of the accuracy of computational methods employed in predicting binding energies, which conforms with the findings of this study, thus validating the methodology. DCC-3014 (vimseltinib), ARRY-382, and BLZ-945 are selective CSF1R inhibitors having the lowest binding energies which elucidates their strong structural affinity and conformational stability with the target kinase. The concomitant IC_50_ values are in concordance with the predicted binding energies which confirm the reliability of the docking methodology elicited.

In conclusion, molecular docking comprises three major steps, namely target identification, lead generation elicited via high throughput screening, and lead optimization. Similar to the results of *in vitro* and *in vivo* analysis, *in silico* analysis cannot be solely used for determining drug efficacy, however, computational modelling can predict the promising potential of leads in a time-efficient and cost-effective manner.

The *in silico* findings evaluate target receptor CSF1R kinase binding sites and subsequently elucidate the downstream effects emerging due to drug inhibition by interacting with particular amino acid residues forming these sites. The authenticity and reliability of the computational docking employed have been refined via docking protocols, as well as by a comparative analysis elicited between *in silico* findings and results reported by peer-reviewed *in vitro* studies as well as clinical trials. The study analysis concludes that Met637-bound drugs with the lowest binding energies show exceptional conformational stability and have lesser off-site adverse effects. Similarly, molecular drug interactions with the hydrophobic pocket of CSF1R formed by Val596, Ala614, Val647, Leu785, and Lys616 help determine pharmacodynamic parameters including potency, dosing and administration. According to this study, the leads DCC-3014, ARRY-382 and BLZ-945 exhibit promising potential as pharmaceutical drugs based on their pharmacokinetic and pharmacodynamic properties analysed computationally.

This study contributes to a better understanding of CSF1R kinase aberrancy in cancer, the promising potential in targeting it, and subsequently reprogramming TAMs to disrupt the tumour microenvironment.

## Abbreviation

3D: 3-dimensional
CSF1: colony stimulating factor-1
CSF1R: colony-stimulating factor-1 receptor
DFG: Asp-Phe-Gly
FDA: Food and Drug Administration
IC_50_: half maximal inhibitory concentration
JMD: juxtamembrane domain
Socs1: suppressor of cytokine signalling family protein 1
STAT: signal transducer and activator of transcription
TAMs: tumor-associated macrophages
TGCT: tenosynovial giant cell tumour
VEGFR2: vascular endothelial growth factor receptor 2

## Declarations

### Copyright

© The Author(s) 2023.
